# Charting the future of *PLOS Medicine*: Priorities for evidence, impact, and equity

**DOI:** 10.1371/journal.pmed.1004691

**Published:** 2025-07-30

**Authors:** Helen Lumbard, Till Baernighausen

**Affiliations:** 1 Public Library of Science, San Francisco, California, United States of America, and Cambridge, United Kingdom; 2 Heidelberg Institute of Global Health, University of Heidelberg, Heidelberg, Germany

## Abstract

As *PLOS Medicine* enters a new chapter, its leadership sets out a bold editorial vision grounded in evidence, impact and equity. In a rapidly evolving global health landscape, the journal reaffirms its commitment to diversity, openness, and actionable science, ensuring research not only reflects the world’s needs, but drives meaningful change.

When *PLOS Medicine* launched just over two decades ago, it did so with a bold and necessary commitment: to make high-quality medical research freely available to all, and to challenge the conventions of medical publishing. That founding mission—centred on openness, integrity, and public purpose—remains as vital as ever. But today, we face a new set of global health challenges that demand renewed ambition, sharper focus, and deeper resolve.

As the journal’s new leadership team—Executive Editor and Editor-in-Chief—we are honoured to take forward this mission at a time of change and opportunity. We bring different but complementary perspectives, united by a shared conviction: that *PLOS Medicine* must lead in ensuring that rigorous, relevant, and equitable research is not only published, but used to improve lives.

The landscape in which we operate is rapidly evolving. Widening health and income inequality, shifting disease burdens, the climate crisis, and digital disruption are reshaping how health research is produced, accessed, and applied. In this context, *PLOS Medicine* must not only reflect the world’s most pressing health questions, but actively shape how those questions are asked, answered, and acted upon.

Medical journals no longer exist simply to disseminate findings. They are active participants in determining which knowledge is prioritized, how it is shared, and who has the opportunity to contribute. In a world marked by geopolitical instability, fragile health systems, and enduring structural injustice, journals carry a responsibility not only to uphold scientific rigor, but to ensure that research represents those who can benefit most and is readily usable in the real world.

At *PLOS Medicine*, our editorial vision is grounded in three core priorities: evidence, impact, and equity.

## Evidence that makes a difference

*PLOS Medicine* has always prioritized high-quality, policy-relevant research across a wide scope of global health and clinical topics, and we will continue to do so. But the health challenges of today and tomorrow require us to look beyond traditional silos. We are expanding our focus to include areas that have long been underrepresented in the medical literature, such as women’s health, mental health, and the complex and growing intersections between climate and health. These are not peripheral concerns; they are foundational to the future of public health and health equity.

We also recognize that generating knowledge is not enough; evidence must be relevant for major disease burdens, and actionable—ideally directly and rapidly—in different health and public health systems. That is why we are committed to strengthening our focus on actionable science. This includes a broader platform for intervention and implementation research, health systems and policy analysis, and research that bridges the gap between discovery and delivery. We aim to spotlight research that not only identifies problems, but also offers solutions, supports scale-up and sustainment, and informs better practice and policy.

## Focusing on meaningful impact

We are committed to understanding and highlighting the real-world effects of the research we publish. While traditional academic metrics offer one view of reach, they do not fully capture the value of research that informs policy, supports public health action, or contributes to better outcomes for communities.

At *PLOS Medicine*, we will continue to prioritize work that has the potential to drive meaningful change, and to explore ways of better communicating the broader influence and use of that research beyond the academic sphere. This includes using our *Policy Forum* and *Health in Action* article types to assess and reflect on the practical implications of research findings, and to highlight how new evidence is shaping implementation, practice, and health policy in real-world settings.

## Equity in research and the real world

Equity begins with inclusion. Research thrives when it draws from the open exchange of ideas, diverse perspectives, and meaningful international collaboration. At *PLOS Medicine*, we are committed to ensuring that the people who shape research—editors, authors, reviewers and readers—reflect the diversity of the communities research is intended to serve.

We believe that a broad range of experiences and expertise strengthens the editorial process and helps ensure that science is more thoughtful, more relevant, and more impactful. We are actively working to diversify our editorial board, create clearer and more inclusive author guidance, and support early-career researchers and those from disciplines or regions historically underrepresented in high-impact journals. Our recent editorial calling for an end to parachute science underscores this commitment and highlights the need for more equitable global research partnerships [1].

Equity also demands openness and transparency in how research is conducted and shared. We are strengthening our policies to improve clinical trial reporting [2], and advancing efforts to make early-stage research more visible through responsible preprint sharing in the health sciences [3].

These efforts are not simply about representation; they are about improving the quality and relevance of what we publish. Our aim is to build a publishing environment in which every contributor—regardless of discipline, geography, or background—has the opportunity to shape the global evidence base.

As a medical journal, we hold a direct lever to promote equity in research and publishing, and through this, we also seek to influence an indirect but powerful lever: the real-world outcomes shaped by the evidence we publish. Research generated by a diverse global community, reflecting a broad range of lived experiences, geographies, and perspectives, is more likely to produce findings that benefit those most in need of health improvements. In contrast, evidence shaped predominantly by researchers from similar backgrounds, using narrow methods in high-income settings, risks reinforcing inequities rather than addressing them.

## Openness with accountability

Undergirding our guiding principles of evidence, impact, and equity, our fully Open Access model remains a cornerstone of this mission. But openness is about more than access; it is also about accountability, transparency, and trust. We uphold high standards for data sharing, reproducibility, and ethical conduct. And we recognise that openness must be accompanied by strong guidance and support for those navigating these expectations, especially for researchers who are newer to open science practices.

We are also working to ensure our publishing processes are as navigable and equitable as the research we share. We are committed to making our systems more transparent and to providing authors, reviewers, and editors with the tools they need to participate fully and fairly in the scientific process.

## Join us in shaping the future

*PLOS Medicine* is entering a new phase - one that reflects both continuity and change within medical publishing. The choices we make now—about what we value, whose voices we amplify, and how we engage with science and society—will shape health outcomes for years to come.

We are grateful to lead this work in partnership with each other, with the *PLOS Medicine* team, and with you: our readers, authors, reviewers, and collaborators. We invite you to join us in charting a future driven by evidence, defined by impact, and grounded in equity.
